# Parallel adaptation to lower altitudes is associated with enhanced plasticity in *Heliosperma pusillum* (Caryophyllaceae)

**DOI:** 10.1111/tpj.16342

**Published:** 2023-06-21

**Authors:** Aglaia Szukala, Clara Bertel, Božo Frajman, Peter Schönswetter, Ovidiu Paun

**Affiliations:** ^1^ Department of Botany and Biodiversity Research University of Vienna Rennweg 14 A‐1030 Vienna Austria; ^2^ Vienna Graduate School of Population Genetics Vienna Austria; ^3^ Austrian Federal Research Centre for Forests (BFW) Unit of Ecological Genetics Seckendorff‐Gudent‐Weg 8 A‐1131 Vienna Austria; ^4^ Department of Botany University of Innsbruck Innsbruck Austria

**Keywords:** latitudinal adaptation, drought tolerance, gene expression plasticity, *Heliosperma pusillum*, local adaptation, parallel evolution

## Abstract

High levels of phenotypic plasticity are thought to be inherently costly in stable or extreme environments, but enhanced plasticity may evolve as a response to new environments and foster novel phenotypes. *Heliosperma pusillum* forms glabrous alpine and pubescent montane ecotypes that diverged recurrently and polytopically (*parallel evolution*) and can serve as evolutionary replicates. The specific alpine and montane localities are characterized by distinct temperature conditions, available moisture, and light. Noteworthy, the ecotypes show a home‐site fitness advantage in reciprocal transplantations. To disentangle the relative contribution of constitutive versus plastic gene expression to altitudinal divergence, we analyze the transcriptomic profiles of two parallely evolved ecotype pairs, grown in reciprocal transplantations at native altitudinal sites. In this incipient stage of divergence, only a minor proportion of genes appear constitutively differentially expressed between the ecotypes in both pairs, regardless of the growing environment. Both derived, montane populations bear comparatively higher plasticity of gene expression than the alpine populations. Genes that change expression plastically or constitutively underlie similar ecologically relevant pathways, related to response to drought and trichome formation. Other relevant processes, such as photosynthesis, rely mainly on plastic changes. The enhanced plasticity consistently observed in the montane ecotype likely evolved as a response to the newly colonized, drier, and warmer niche. We report a striking parallelism of directional changes in gene expression plasticity. Thus, plasticity appears to be a key mechanism shaping the initial stages of phenotypic evolution, likely fostering adaptation to novel environments.

## Significance Statement

In early stages of adaptation to hotter and drier habitats, an alpine plant in the carnation family shows increased expression plasticity at ecologically relevant genes.

This paper investigates two contrasting hypotheses regarding the role of phenotypic plasticity during evolution of alpine/montane plant ecotypes.

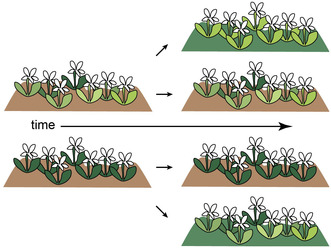

## INTRODUCTION

How phenotypic divergence between conspecific populations arises, leading to local adaptation, stable differentiation, and ultimately speciation, is a central question in evolutionary biology. A property of organisms, which can determine population differentiation across heterogeneous environments, is plasticity, that is, the capacity of a genotype to change its phenotype upon exposure to differing environmental conditions (Schlichting & Pigliucci, 1998). It is still poorly investigated in which conditions plasticity impedes or promotes long‐term phenotypic change, and what are the mechanisms translating short‐term environmental responses into long‐term evolutionary states (Healy & Schulte, 2015; Sommer, 2020; Stearns, 1989; Stotz et al., 2021).

Despite being a necessary property to survive in variable environments, especially for sessile organisms such as plants, plasticity may be inherently evolutionarily costly as soon as the population has reached an adaptive optimum (DeWitt et al., 1998; Pál & Miklós, 1999). Moreover, some authors advanced the hypothesis that plasticity might reduce the power of natural selection, representing a dead end of evolution (Charlesworth et al., 1982). In stark contrast, phenotypic plasticity has been proposed as a primary object of selection by others (Levis & Pfennig, 2016; Stotz et al., 2021; Waddington, 1942), who called for a reconsideration of its importance for the evolutionary process. Adaptive benefits of plasticity have been in certain circumstances documented, for example, for traits related to biotic responses (Auld & Relyea, 2011) and abiotic stress (Dudley & Schmitt, 1996; Nicotra et al., 2015; Sole‐Medina et al., 2022; Stotz et al., 2021). However, plasticity of some traits can also be neutral or even maladaptive when environmentally induced phenotypes have reduced fitness (Arnold, Kruuk, & Nicotra, 2019; Dorn et al., 2000; van Kleunen & Fischer, 2005). How and under which conditions plasticity supplies the phenotypic variation later refined by selection remains debated (Arnold, Nicotra, & Kruuk, 2019; Flatscher et al., 2012; Fox et al., 2019; Levis & Pfennig, 2016; Wund, 2012).

Plasticity has been linked to local adaptation on rare occasions. Corl et al. (2018) showed, for example, how plasticity at genes controlling skin coloration of the common side‐blotched lizard facilitated colonization of dark‐soil environments and suggested that genetic changes in the same genes were shaped by natural selection refining a pre‐existing plastic phenotype. Similarly, Levis et al. (2018) reported that in spadefoot toad tadpoles adaptive novelty can arise from pre‐existing plasticity in diet‐related morphological and molecular features. However, the study also uncovered diet‐induced maladaptive plasticity affecting not only mouthpart formation but also expression of a diet‐relevant gene. In *Silene uniflora*, ancestral plasticity has been shown to move expression of genes important to adaptation closer to optimum values (Wood et al., 2023). Selection appears to promote enhanced plasticity in certain conditions but not in others, as shown, for example, in the waxy bluebell *Wahlenbergia ceracea* (Campanulaceae; Nicotra et al., 2015). In this plant, low‐elevation populations show enhanced temperature‐induced plasticity, which was found to be more often adaptive compared to populations from higher elevations.

**Figure 1:**
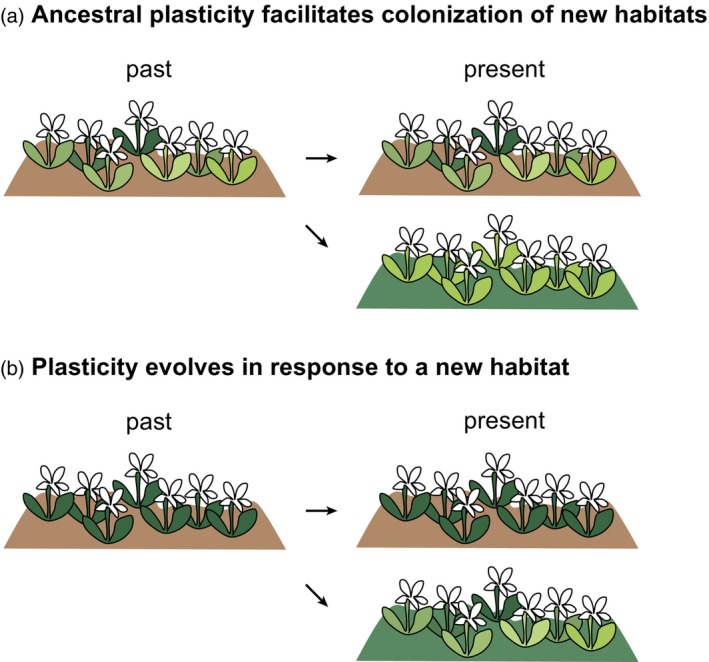
The schemes of two contrasting hypotheses regarding the role of phenotypic plasticity during evolution of different ecotypes. The heterogeneity in plant color symbolizes here the degree of plasticity at different stages, whereas brown and green backgrounds indicate an ancestral and a derived niche, respectively. Other aspects, for example, the amount of genetic variation in the population, its size, or the temporal and spatial environmental heterogeneity, are not taken into account in these simplified, hypothetical scenarios. (a) Pre‐existing plasticity in an ancestral population (e.g., in a heterogeneous environment) facilitates the colonization of new habitats. The phenotype is ultimately refined in the newly occupied habitat, where plasticity could be lost over time, due to genetic assimilation. (b) The ancestral population bears little plasticity, which evolves in response to a newly colonized environment. This scenario has been referred to as “plasticity‐led evolution” (Levis & Pfennig, 2016; Schwander & Leimar, 2011) or Baldwin effect (Healy & Schulte, 2015).

Evidence from natural study systems is required to clarify under which conditions plasticity is favored or hindered by evolution. A meta‐study by Barley et al. (2021) quantified thermal acclimation capacity across 19 species including arthropods, mollusks, and chordates, showing that within species, marginal populations experiencing the highest thermal conditions had decreased plasticity and acclimation capacity. A negative relationship between plasticity and adaptation to heat extremes was also found in laboratory experiments (Kelly et al., 2017; Sasaki & Dam, 2021). These results suggest that there is a trade‐off for plasticity at ecological extremes (Chevin & Hoffmann, 2017), such that extreme environments appear to favor phenotypic robustness through *canalization* (Waddington, 1942). On the other hand, evolve and resequence studies on *Drosophila melanogaster* have shown that after 60 generations of adaptation to hot temperatures, 75% of genes evolved higher plasticity (Mallard et al., 2020), suggesting that adaptation to novel environments leads to an initial increase in plasticity. Altogether, it is still unclear whether plasticity precedes and facilitates migration to novel environments, or whether the initial exposure to novel conditions rather fosters increased plasticity (Figure 1), followed by a progressive loss of plastic potential through *genetic assimilation* (Ehrenreich & Pfennig, 2016) toward adaptation. These two scenarios are not mutually exclusive, but they would be involved at different stages of colonization and adaptation; both suggest that plasticity plays an important role during early phases of adaptation.

Here, we investigate gene expression plasticity versus genetically encoded expression differentiation during the initial stages of parallel divergence in the plant *Heliosperma pusillum* (Waldst. and Kit.) Rchb. (Caryophyllaceae; James et al., 2023; Szukala et al., 2023; Trucchi et al., 2017). This species forms altitudinal ecotypes previously shown to bear cross‐generation phenotypic differentiation (Bertel et al., 2018; Bertel, Hülber, et al., 2016). The alpine ecotype is widely distributed from the Spanish Cordillera Cantabrica to the Romanian and Ukrainian Carpathians, inhabiting wet screes, rock faces, and open grasslands above the timberline, typically at elevations between 1400 and 2300 m. By contrast, the montane ecotype (previously referred to as *H. veselskyi* Janka; Frajman & Oxelman, 2007; Bertel, Buchner, et al., 2016; Bertel et al., 2017, 2018; Trucchi et al., 2017) is known to be restricted to the south‐eastern Alps, being represented by typically isolated and very small populations, mostly below overhanging cliffs in poor light conditions and shaded from rain in the montane belt (500–1300 m). Noteworthy, previous genomic and transcriptomic studies (Szukala et al., 2023; Trucchi et al., 2017) have demonstrated, among others with coalescent methods, that pairs of geographically clustered montane and alpine ecotypes in the south‐eastern Alps have diverged at least four times independently, representing a case of parallel evolution and can thus be regarded as natural evolutionary replicates.

**Figure 2:**
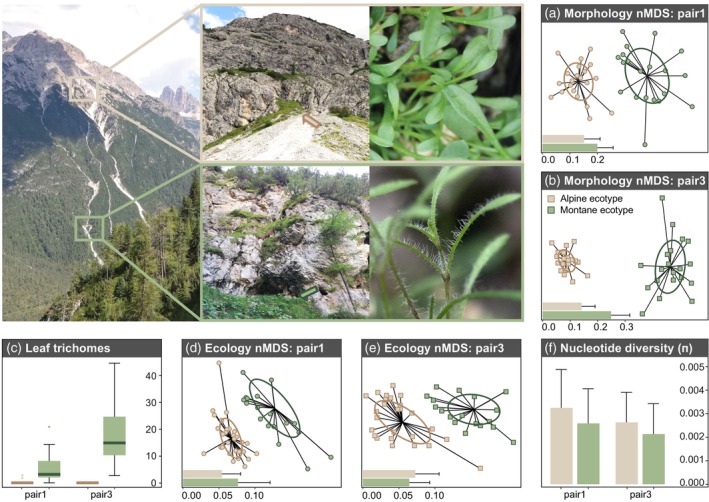
Summary of within‐population morphological, ecological, and genetic diversity within the two population pairs of *Heliosperma pussilum* investigated here, drawn from previously published data (Bertel et al., 2018). Green‐ and brown‐filled elements represent the montane and alpine ecotypes, respectively, while circles and squares represent pair 1 and 3, respectively (i.e., each representing an independent instance of ecotype divergence as explained in Material & Methods). Photographs of typical localities and representative plants by A. Szukala and M. Eriksson. (a,b) Non‐metric multidimensional scaling (nMDS) showing increased morphological variability in the montane ecotype among 16 morphometric characters measured on 20 individuals per population, that were grown in their natural habitat, plotted for each ecotype and ecotype pair using the data from Bertel et al. (2018). Confidence ellipses around treatment centroids represent the standard deviation of the measurements of the respective group. The bar charts on the lower left corner are the mean and SD of the dissimilarity matrices based on Bray–Curtis distances for each population. (c) Pronounced variation in the amount of glandular trichomes measured in the montane ecotype, as compared to the alpine, potentially suggestive of increased plasticity in the former. The boxplots are drawn from data of Bertel et al. (2018). (d,e) Non‐metric multidimensional scaling (nMDS) of ecological differentiation. Ordinations are based on dissimilarity matrices of mean Landolt indicator values of the accompanying vegetation, that is, all species growing within a circular area of 0.2 m radius centered on 30 individuals per population, drawn from data of Bertel et al. (2018). Landolt indicator values are numbers ranging from 1 to 5, that are defined in the literature (Landolt et al. 2010) and characterize the ecological requirements of species in terms of important ecological parameters, that is, temperature, continentality, light, soil moisture, soil reaction, soil nutrients, soil humus content, soil aeration, soil moisture variability. (f) Estimates of within‐population genetic diversity (as nucleotide diversity, π) in each pair, calculated using RNA‐seq data from Szukala et al. (2023).

The lack (alpine) or presence (montane) of a dense indumentum with long multicellular sticky glandular trichomes on stem and leaves is the most divergent morphological trait between the ecotypes (Figure 2; Bertel, Buchner, et al., 2016; Bertel et al., 2017, 2018). The morphological divergence is most strongly correlated with temperature and soil humidity differences between the two altitudinal sites, whereas humidity and light availability show higher temporal variability at the montane sites compared to the alpine ones (Bertel et al., 2018). Along the same lines, montane traits such as multicellular trichomes and physiological response to low light (Bertel, Buchner, et al., 2016) typically show greater variability across and within montane populations (Figure 2a–c; Bertel et al., 2018). This variability is likely at least in part due to plasticity, given that reduced genetic variation was found in the small and strongly localized montane populations (Figure 2f; Szukala et al., 2023; Trucchi et al., 2017). Reciprocal transplantation experiments performed at the native altitudinal sites (Bertel et al., 2018) demonstrated a home‐site fitness advantage of each ecotype in terms of establishment success (i.e., measured as the proportion of plants alive 1 year after germination). Higher survival rates of either ecotype in its respective native environment are strong indicators that the morphological and physiological differentiation has adaptive value.

Here, we investigate phenotypic plasticity during the initial phases of recurrent ecotype divergence in *H. pusillum* (Trucchi et al., 2017; Szukala et al., 2023), in the context of our two hypotheses (Figure 1). We quantify gene expression divergence in two parallely evolved ecotype pairs upon reciprocal transplantations at natural growing sites, to identify (i) genes that diverge in expression between the two ecotypes regardless of the growing environment (*constitutive component of gene expression divergence*), or (ii) genes that change their expression plastically as a function of the environment (*plastic component of gene expression divergence*), and (iii) differences in the amount of gene expression plasticity that evolved in parallel. To reinforce our interpretation of the observed patterns in light of the hypotheses presented in Figure 1, we investigate the amount of private genetic variation and minor allele frequencies in the ecotypes. Finally, we discuss how plasticity might pave the path towards adaptation during the early stages of ecological divergence.

## RESULTS

### Gene expression differences are driven by origin (ecotype pair) and ecotype divergence

**Figure 3:**
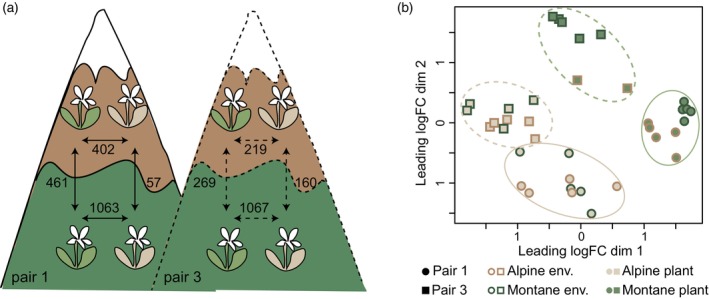
Study design and summary of its results. (a) Setup of the reciprocal transplantations between alpine (symbolized with brown areas of the mountains) and montane sites (shown with dark green areas). Brown plants represent the alpine ecotype and green plants the montane one. The numbers represent differentially expressed genes in different comparisons (adjusted *P* < 0.05). For simplicity, the two ecotype pairs are here shown on different mountains, but they have been both reared together at the native localities of pair 1. (b) Multidimensional scaling plot of distances between gene expression profiles of individuals of the two ecotypes grown at different altitudes. Circles and continuous lines represent the ecotype pair 1, and squares and dashed lines the ecotype pair 3. Green‐ and brown‐filled symbols show the montane and alpine ecotypes, respectively, while green and brown symbol margins represent the low and high growing sites, respectively.

To be able to investigate the interaction between altitude and gene expression, we isolated RNA from leaves of two ecotype pairs grown at either the alpine or the montane natural sites (Figure 3a). The two altitudinal niches are characterized by stark differences in average and amplitude of temperature, water, and light availability (Bertel et al., 2018) but also by distinct biotic environments (Bertel et al., 2018; Trucchi et al., 2017). After filtering out genes with low normalized expression counts, we searched a total of 15 591 genes for genetically versus environmentally driven expression divergence between ecotypes and environments. Multidimensional scaling (MDS, Figure 3b) and principal component analysis (PCA, Figure s1) of normalized read counts showed that gene expression clusters the samples by ecotype pair (i.e., pair 1 shown with circles in Figure 3b versus pair 3 shown with squares; in Figure s1 PC1 explaining 17.0% of variance), as well as by ecotype (i.e., alpine shown with brown‐filled symbols in Figure 3b versus montane shown with green‐filled symbols; in Figure s1 PC2 explaining 10.4% of variance). The variable growing environment explained less variance of the data (montane environment shown with symbols with green margins versus alpine environment shown with symbols with brown margins; in Figure s1 PC5 explaining 4.7% of variance). This clustering shows that the locality of origin of each plant and the divergence between ecotypes explain most of the expression patterns recovered.

**Figure 4:**
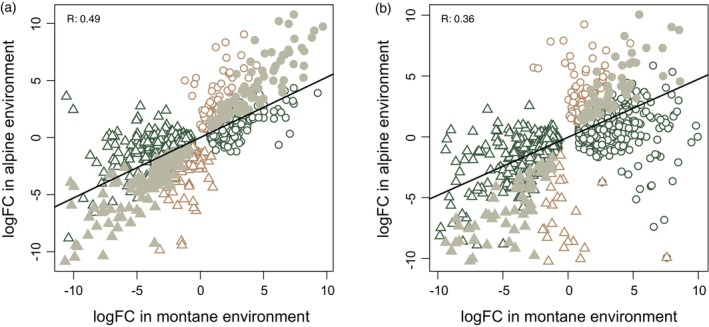
Differentially expressed (DE) genes between ecotypes in different environments. The X‐ and Y‐axis show log fold‐change values for genes with differential expression between ecotypes when these are grown in the montane (green and gray symbols) and alpine (brown and gray symbols) environment, respectively, for ecotype pair 1 (a) and 3 (b). Triangles and circles represent genes under‐ and overexpressed in the montane ecotype compared with the alpine, respectively. Gray‐filled symbols show genes with stable, non‐plastic expression divergence between ecotypes, independent of the environment. The black line shows the correlation between gene expression changes in the montane and alpine environments.

We observed that gene expression differences between ecotypes were more pronounced in the montane environment compared to the alpine in both pairs analyzed (Figures 3 and 4). In the montane environment, we found 1063 differentially expressed (DE) genes between the two ecotypes of pair 1 (652 under‐ and 411 overexpressed in the montane ecotype compared to the alpine one) and 1067 DE genes between the two ecotypes of pair 3 (483 under‐ and 584 overexpressed in the montane ecotype compared to the alpine one; green and gray symbols in Figure 4). By contrast, significantly fewer genes were found to be DE in the alpine environment (brown and gray symbols in Figure 4) with 402 DE genes between ecotypes in pair 1 (246 under‐ and 156 overexpressed in the montane ecotype compared to the alpine one) and 219 in pair 3 (83 under‐ and 136 overexpressed in the montane ecotype compared to the alpine one). Despite these differences in expression patterns observed when comparing the situation between the growing environments, in both ecotype pairs the expression patterns at either altitude were positively correlated (Spearman's correlation 0.49 and 0.36 in pairs 1 and 3, respectively, Figure 4), implying that the direction of gene expression changes between ecotypes is stable upon environmental change.

### Constitutive evolutionary changes in gene expression

Among the DE genes 216 (significantly more than the 27 genes expected by chance; hypergeometric *P* < 1e‐147) and 118 (significantly more than the 14 genes expected by chance; hypergeometric *P* < 2.6e‐79) genes (gray symbols in Figure 4) were always DE between ecotypes in the same direction in pairs 1 and 3, respectively, regardless of the growing environment. These genes that do not show significant environmental sensitivity represent constitutive expression divergence and are most likely relevant in shaping stable trait differences between ecotypes. Moreover, the genes with constitutive expression divergence appeared to shape a considerable proportion of expression differences in the alpine conditions—that is, representing consistently ca. 54% of overall expression differences between ecotypes in this environment in both pairs (compared to 20% and, respectively, 11% in the montane environment). Among the constitutive genes identified in each pair, 26 genes (Table s1) were shared by both ecotype pairs (significantly more than two genes as expected by chance; hypergeometric *P* < 3e‐24). Finally, eight of these genes (Table s1) were also found to be DE in both ecotype pairs in a non‐native, common garden environment in a previous study (Szukala et al., 2023), despite the different growing conditions and developmental stage.

### Environmentally sensitive gene expression

**Figure 5:**
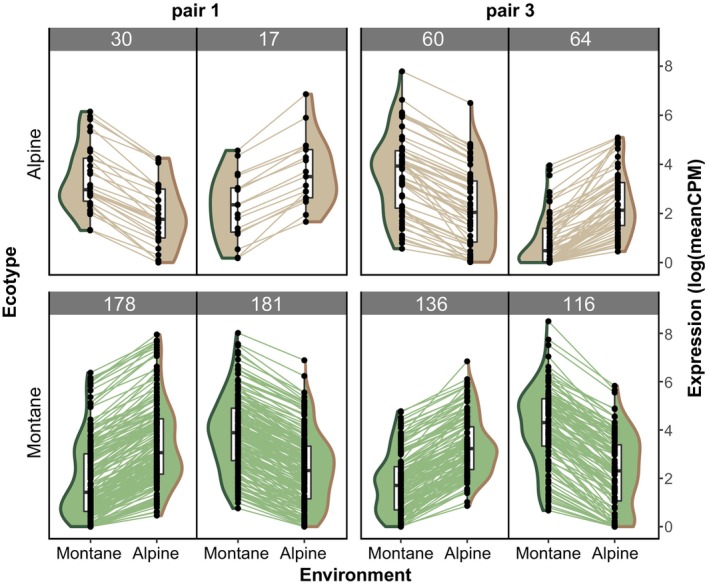
Genotype‐by‐environment interactions are more pronounced in the montane ecotype, as exemplified by environmentally driven gene expression changes. Brown‐ and green‐filled violin plots represent the montane and alpine ecotypes, whereas green and brown violin plot margins represent the montane and alpine environment, respectively. Genes DE (adjusted *P* < 0.05, logFC >1.5) when alpine (upper row) or montane (lower row) ecotypes are grown at different altitudes are reported. The numbers on top of each plot give the number of genes in the respective category. Each dot represents the average expression of a gene in a given environment, while lines connecting two dots show the expression change of a particular gene at different altitudes.

We also looked for environmentally induced expression changes within each ecotype, when these are grown at different elevations (i.e., G × E interaction). We found 461 (pair 1) and 269 (pair 3) DE genes in the montane ecotype versus 57 (pair 1) and 160 (pair 3) DE genes in the alpine ecotype that were explained by the variable “altitude” of the generalized linear model (Figures 3a and 5). These results suggest that gene expression in the montane ecotype is strongly modified depending on the altitude, implying more pronounced expression plasticity than in the alpine ecotype. This pattern was particularly pronounced in pair 1. In both pairs, the amount of genes showing significant expression plasticity in the montane ecotype is more than double the amount of constitutive DE genes shaping ecotype differentiation. The amount of plastic DE genes shared by both ecotypes (i.e., six) was close to random expectation (hypergeometric *P* = 0.04). We did not observe a clear pattern of down‐ or upregulation of gene expression in the non‐native environment that was consistent across both pairs. Also, in both ecotypes, the amount of down‐ versus upregulated genes in the non‐native environment was similar.

### Biological significance of constitutive DE genes

The 26 genes consistently DE between ecotypes regardless of the environment and shared by both ecotype pairs are reported with GO term annotations in Table s1. The genes overexpressed in the montane ecotype (positive logFC in both pairs) are involved in response to salt stress and water deprivation (*BSK11*, *CER1*), epigenetic regulation of gene expression by methylation (*DNMT2*), and protein phosphorylation (*LRR‐RLK*, *RPS20B*), while underexpressed genes in the montane ecotype (negative logFC in both pairs) play roles in immunity (*FUC1*, *At4g35733*, *CYP83B1*) and enhanced drought and salt tolerance (*CLB*, see de Silva et al., 2011 for increased salt tolerance in knock‐out mutants of *Arabidopsis thaliana*).

**Figure 6:**
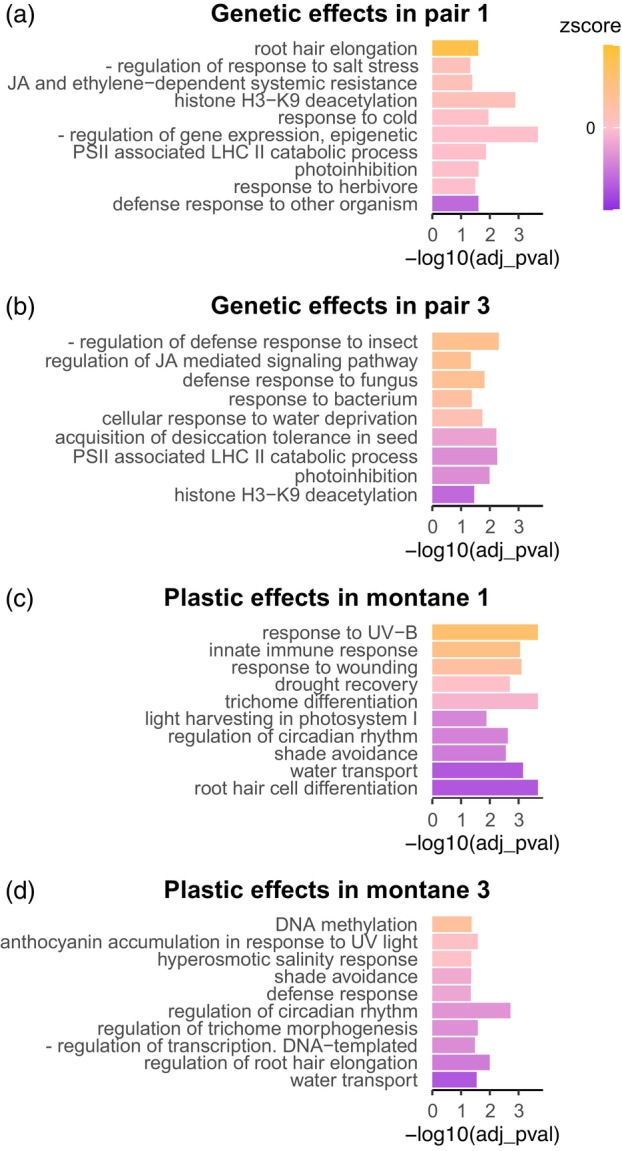
GO terms enrichment (biological processes) of constitutive versus plastic DE genes. Enriched functions in constitutive expression differentiation between ecotypes in pairs 1 (a) and 3 (b). Enriched functions in genes changing their expression plastically between altitudes in the montane ecotypes 1 (c) and 3 (d). Each bar corresponds to a GO term (Y‐axis), while the size of the bars corresponds to the significance of the enrichment (adjusted *P* < 0.05). The color scale represents the z‐score, which is computed based on the logFC of expression of each gene underlying a specific GO term with orange shades corresponding to overexpression in the montane ecotype (a and b) or the montane environment (c and d), and violet shades indicate an underexpression in the montane ecotype (a and b) or the montane environment (c and d). GO terms reported were selected because of their ecological relevance from a larger list of significant GO terms, fully reported in Tables S2–S5. JA—jasmonic acid; LHC—light‐harvesting complex; PSII—photosystem II.

Additionally, we performed GO terms enrichment of the genes that were consistently DE between ecotypes regardless of the environment (i.e., *constitutive DE genes*) but specific for each ecotype pair separately, to clarify whether different sets of constitutive genes do underlie similar functional networks and adaptive responses (Figure 6a,b, Tables S2 and S3). In both pairs, we found significant enrichment (adjusted *P* < 0.05, Fisher's exact test) of response to water deficit and salinity, as well as responses to ABA, probably related to stress responses (Figure 6a,b). Despite the convergence of the enriched GO terms, the number of genes underlying each term, as well as the z‐score exemplifying the overall expression direction change differed in the two evolutionary replicates (Wolfe et al., 2021). Ecologically relevant enriched functions that were not shared by the two pairs have also been identified. In pair 1, DE genes overexpressed in the montane ecotype were enriched for root hair elongation (i.e., a pathway representative also for multicellular trichome development in plants) and epidermal cell differentiation, as well as stomatal closure and negative regulation of gene expression (Figure 6a). In pair 3, genes overexpressed in the montane ecotype were involved in negative regulation of defense responses, as well as jasmonic acid‐mediated signaling (Figure 6b). The full lists of GO terms enriched in pair 1 and, respectively, pair 3 for genes constitutively DE between ecotypes are reported in Tables S2 and S3.

### Biological significance of plastic DE genes

We report in Figure 6c,d, and Tables S4 and S5 the GO terms enriched for the genes changing expression plastically in the montane ecotype of both pairs, and in Tables S6 and S7, the GO enrichment of the genes changing expression plastically in the alpine ecotype. Terms enriched in plastic DE genes in the montane ecotype were similar among the two ecotype pairs, including response to high light intensity (such as photosynthesis, anthocyanin biosynthesis, response to UV‐B and shade avoidance), trichome or root hair differentiation, regulation of circadian rhythm, response to salinity and water transport, and regulation of transcription and methylation. Plastic differential gene expression in the montane ecotype tended to be characterized by pronounced downregulation in the alpine environment in both pairs (Figure 6c). The direction of expression changes underlying the same function was often inconsistent between pairs, likely depending on the function of different genes affecting the same pathway (Figure 6c,d). As mentioned above, the alpine ecotype showed reduced plasticity of gene expression compared to the montane one, especially in pair 1. Despite the lower number of DE genes underlying enriched function, we found some similar functions to be enriched as in the montane ecotype (e.g., regulation of circadian rhythm and response to light).

### Signatures of ecotype divergence in DE genes

We looked for signatures of ecotype divergence in DE genes, defined as genes carrying loci falling in the top 5% of the fixation index (*F*_ST_) distribution. We found that in both ecotype pairs DE genes between ecotypes at each altitude included a proportion of divergence outliers higher than chance expectations, while plastic DE genes did not (Table S8). More specifically, in pair 1, we found that of the DE genes between ecotypes detected in the montane and alpine environment, respectively, 20 (*P* = 3.15e‐5) and 11 (*P* = 8.99e‐5) DE genes also carried divergence outliers. Five among these genes were constitutive DE genes, that is, differentially expressed at both altitudes. In pair 3, we found that of the DE genes between ecotypes detected in the montane and alpine environment, respectively, 13 (*P* = 0.0062) and 4 (*P* = 0.031) DE genes also carried divergence outliers, including three constitutive DE genes. Among these divergence outliers being also DE genes, we report some DNA‐binding transcription regulators (Table S8, e.g., the Scarecrow‐like protein 5 and the DNA‐binding bromodomain‐containing protein found to be constitutively DE in pair 1, and the Transducin/WD40 repeat‐like superfamily protein found to be constitutively DE in pair 3) that likely play a major role in ecotype divergence as key regulators of a number of genes. None of the 26 constitutive DE genes shared by both pairs contained divergence outliers. In plastic genes of pair 1, we identified three (*P* = 0.22) and zero DE genes carrying divergence outliers in the montane and alpine ecotypes, respectively, while in pair 3, one (*P* = 0.33) and two (*P* = 0.17).

### Population‐wise private alleles

After calling and filtering high‐quality private single nucleotide polymorphisms (SNPs) in each one of the four studied populations we observed an excess of private polymorphisms in the alpine populations compared to the montane ones (Figure S2a and S3). Additionally, we observed that minor allele frequencies (MAFs) among private alleles tend to be high (i.e., mean(MAF) > 0.2 in all populations, Figure S2b), suggesting that a majority of them did not accumulate during recent population expansion. We also observed that MAFs of private alleles tend to be similar between montane and alpine populations, suggesting that private variation is not strongly affected by opposite trends in the evolution of the effective population size between ecotypes (i.e., larger alpine populations versus smaller montane ones). Taken together, these results are consistent with previous molecular results (Frajman & Oxelman, 2007; Szukala et al., 2023; Trucchi et al., 2017) and suggest that the montane ecotypes are derived from the alpine populations of the species.

## DISCUSSION

To date, a handful of studies have investigated the evolution of plasticity during the early stages of adaptation in natural (Corl et al., 2018; Levis et al., 2018; Passow et al., 2017; Scoville & Pfrender, 2010; Wood et al., 2023) or experimental populations (Brennan et al., 2022; Huang & Agrawal, 2016; Mallard et al., 2020; Sikkink et al., 2019). Here, we have investigated both constitutive and plastic changes in gene expression of altitudinally segregated ecotypes upon reciprocal transplantations in their natural growing sites.

Our results suggest that a combination of constitutive expression divergence and different degrees of expression plasticity underlying the same ecologically relevant functions plays an important role in shaping ecotype divergence. More specifically, the montane ecotype is characterized by enhanced expression plasticity, in agreement with the observed increase in variance in morphological traits in the montane ecotype compared to the alpine ecotype (Figure 2a‐c; Bertel et al., 2018). Our results are consistent between the two ecotype pairs analyzed pointing to a high degree of parallelism in the evolution of expression plasticity in our system. Plasticity might indeed be beneficial in the stressful montane niche under overhanging cliffs, which is characterized by highly variable light and water availability, longer periods of drought, and comparatively high average temperatures (Bertel et al., 2018).

As a consequence of the enhanced plasticity in the montane ecotype, the expression profiles of the ecotypes were more similar in the alpine environment, while they differed strongly in the montane one (Figure 3a), except for a minor proportion of constitutive expression changes. Interestingly, constitutive DE genes showed signatures of selection as *F*_ST_ outliers. Such outlier constitutive DE genes included transcription factors that are expected to differentially affect transcription levels in a large cohort of genes, and may therefore be responsible for the observed differences between plasticity of the two ecotypes. Similar to our results, enhanced phenotypic plasticity in low‐elevation individuals compared with high‐elevation ones was found in *Wahlenbergia ceracea* by Nicotra et al. (2015). In this species, higher plasticity in low‐elevation plants was shown to be adaptive, whereas plasticity in high‐elevation plants was more likely to be maladaptive. In the same study, higher epigenetic diversity in response to growth temperature detected in seedlings from low elevation suggested a role for DNA methylation in shaping adaptive plastic responses. Possibly in line with these results, our GO terms enrichments (Figure 6, Tables S2–S6) showed that both constitutive gene expression divergence, as well as gene expression plasticity are enriched for epigenetic processes (e.g., histone H3‐K9 deacetylation and methylation in constitutive and plastic DE genes, respectively). However, a previous proof‐of‐concept study did not reveal significantly different within‐ecotype levels of genome‐wide DNA methylation variation between the two ecotypes (Trucchi et al., 2016).

By contrast, the alpine ecotype bears a lower plastic potential of gene expression. This ecotype experiences extreme environmental factors (e.g., higher amplitude of seasonal temperature fluctuations, low temperatures, and enhanced solar irradiation), consistent with the hypothesis that extremes lead to enhanced robustness of gene expression and lack of plasticity (Chevin & Hoffmann, 2017; Lande, 2009; Wood et al., 2023). The inability of the alpine ecotype to react plastically to the altitudinal transplantation is possibly consistent with a (albeit not significantly) lower establishment success of alpine plants transplanted to the montane environment, compared with transplanted plants of montane origin at alpine elevation (Bertel et al., 2018).

As many alpine plant species (Giesecke et al., 2017), *H. pusillum* likely migrated upslope after the Last Glacial Maximum (LGM) tracking its specific alpine conditions; the few montane populations present today could represent relicts of the original LGM populations. The alpine ecotype was resolved as ancestral in the study of Frajman and Oxelman (2007) and the time of divergence between ecotypes was estimated around LGM (Trucchi et al., 2017), albeit older in another study (Szukala et al., 2023). Accordingly, our analyses detected a higher amount of private genetic variation in the alpine ecotype, which can be used as a proxy for the ancestral state (Paun et al., 2008; Schönswetter & Tribsch, 2005). The relict montane populations likely adapted to the specific niche under overhanging cliffs with a lack of competition but high levels of abiotic stress (Davis, 1951; García & Zamora, 2003; Minuto et al., 2012), as these progressively became warmer and the original alpine habitats at low elevations disappeared due to the advancement of forests during the Holocene. The alpine ecotype, on the other hand, enlarged its distribution range throughout the southern European mountain ranges, where its habitats are abundant. Despite both habitats likely differing from the ancestral environment preceding ecotype divergence, we suggest that the alpine niche is more likely to resemble the ancestral one with regard to temperature and humidity, as well as biotic interactions. Under this scenario, the montane ecotype has enhanced expression plasticity as a consequence of exposure to new conditions in the montane environment, starting from an ancestral state, in which plasticity was lacking (Figure 1b). Alternatively, the alpine ecotype might have lost ancestral plasticity through canalization (Figure 1a). Although this second hypothesis appears less likely, it cannot be ruled out with certainty.

Despite some differences in the magnitude of the patterns found, our results are consistent between the two ecotype pairs analyzed, which represent independent instances of ecotype formation (Szukala et al., 2023; Trucchi et al., 2017), and can therefore be considered natural evolutionary replicates. It is important to notice that we could sample only two surviving biological replicates of the montane ecotype from pair 3 transplanted to the alpine site. Interpretations of the results regarding this group should therefore be considered with caution. Still, while differences in the absolute numbers observed between ecotype pairs might have been driven by these differences in sampling density, the overall patterns of evolutionary versus plastic expression changes should not be affected severely.

Adaptation to new environments can produce a large shift in the degree of plasticity. Here, we reported enhanced plasticity in the montane environment in at least two independent divergence events. Our results align with experimental studies showing that adaptation to novel conditions (e.g., high temperature) increases gene expression plasticity (Brennan et al., 2022; Mallard et al., 2020). Moreover, exposure to abiotic stress, such as drought, salinity, and heat, induced high gene expression plasticity in *Brachypodium distachyon* (Priest et al., 2014). Like in *Heliosperma*, convergence in the evolution of plasticity was found in two parallelly evolved zinc‐tolerant lineages of *Silene uniflora* (Wood et al., 2023). Nevertheless, zinc‐tolerant *Silene*‐derived populations appeared to have decreased plasticity due to genetic assimilation of ancestral plasticity. Future studies should aim to directly assess whether expression plasticity in the montane ecotype of *Heliosperma pusillum* changes the phenotype deterministically in such a way that fitness is increased, in order to drive stronger conclusions about the impact of natural selection on plasticity in this system. Understanding the importance of phenotypic plasticity for fast adaptation to abiotic stress is very timely also for crops and breeding (Dalal et al., 2017; Fox et al., 2019; Shao et al., 2007).

We found that over 50% of the genes DE between ecotypes in the alpine environment were also DE in the montane environment, implying that an important part of expression divergence in the alpine environment is driven by genetic change, while a major additional proportion of divergence in the montane environment is plastic. Consistent with a previous investigation of ecotype‐specific gene expression profiles in a common garden (Szukala et al., 2023), we found a limited, even if still significant amount of constitutive DE genes shared by the two ecotype pairs. Interestingly, eight among these 26 genes were previously found to be DE in both pairs in the common garden experiment of Szukala et al. (2023), even if the seeds were collected in a different year and RNA was extracted from leaves at a different developmental stage and in a different environment. The low overlap of DEGs between pairs confirms the previously observed heterogeneity of DE genes in parallely evolved ecotype pairs (Szukala et al., 2023), suggesting redundant adaptive solutions to cope with altitudinal differentiation. Heterogeneity was recovered also in the direction of gene expression changes between ecotypes: while in pair 1 underexpression in the montane ecotype was more frequent, in pair 3 the opposite pattern was observed. Notably, we recovered similar biological functions enriched for constitutive DE genes, especially related to (a‐)biotic defense responses, such as herbivory, temperature, water deprivation, and salt stress (note that the last two stressors are functionally strongly interconnected; Ma et al., 2020), light availability, and epigenetic regulation, despite the limited overlap of specific constitutive genes. Similar functions related to the morphological (i.e., differences in hairiness) and ecological (i.e., differences in temperature, and water and light availability) divergence of the populations were underlied by both plastic and constitutive gene expression divergence.

In summary, the comparison of gene expression patterns between ecotypes upon reciprocal transplantations provided insights into the relative roles of expression plasticity and evolution in shaping gene expression divergence in nature. Similar to precedent studies (McCairns & Bernatchez, 2010; Narum & Campbell, 2015), our findings point to an intricate interaction of evolutionary changes and plasticity, and to an important role of expression plasticity favoring the colonization of novel habitats during early stages of divergence. Future studies should aim for a better understanding of the regulatory patterns behind plasticity and its role in shaping adaptation.

## EXPERIMENTAL PROCEDURES

### Reciprocal transplantations and plant material

Reciprocal transplantations were carried out in 2017 in Lienzer Dolomiten, Kärnten (Austria; alpine site: 46.762 N 12.877 E, 2055 m; montane site: 46.774 N 12.901 E, 790 m). Seeds were collected from wild populations of both ecotypes at these two localities and in the Puez‐Geisler region, Trentino‐Südtirol/Alto Adige (Italy; alpine site: 46.601 N 11.768 E, 2290 m; montane site: 46.564 N 11.77 E, 1690 m). We use the same acronyms as in Bertel et al. (2018) and Szukala et al. (2023), and name the ecotype pair from Puez‐Geisler as pair 1, and that from Lienzer Dolomiten as pair 3, to facilitate comparisons between studies. Seeds were first germinated in a common garden in the Botanical Garden of the University of Innsbruck, Austria, and young seedlings were then transferred to the transplantation sites and grown for one season before sampling leaves in early autumn 2017. This approach was necessary, as transplantation trials attempting germination directly at the native sites showed insufficient and erratic germination rates, especially in the dry montane habitats (Bertel et al., 2018).

Our approach included initially 160 individuals, and originally aimed to investigate with RNA‐seq a total of 40 individuals: two ecotypes × two pairs × two elevations × five individuals. More specifically, 5 individuals of each ecotype and each original pair (i.e., pair 1 and 3, summing up to 10 individuals per ecotype in total) were grown at each elevational site (i.e., montane and alpine), summing up to 20 individuals per ecotype grown in this experiment. However, due to the death of individuals during the course of the experiment (i.e., the alpine site experienced pronounced damage by chamois, among others), only two individuals remained available for the group of montane individuals from pair 3 transplanted to the alpine site. For this reason, our final analyses comprise a total of 37 individuals with five biological replicates per group and one group with only two biological replicates. When more than five accessions survived per group, five individuals were chosen randomly.

### Library preparation and sequencing

Fresh vegetative shoots from transplanted and native plants were fixed in RNAlater (Sigma–Aldrich, Vienna, Austria) on the same day and time of the day (ca. 2:00 pm) and stored at −80°C until further processing. Total RNA was extracted from *ca* 90 mg leaves using the mirVana miRNA Isolation Kit (LifeTech, Vienna, Austria) following the manufacturer's instructions, and it was further depleted of residual DNA with an RNase‐Free DNase Set (Qiagen, Vienna, Austria) and of the abundant ribosomal RNA by using a Ribo‐Zero rRNA Removal Kit (Illumina, Vienna, Austria). RNA was quantified with a NanoDrop2000 spectrophotometer (Thermo Scientific, Vienna, Austria), and its quality was assessed using a 2100 Bioanalyzer (Agilent, Vienna, Austria). NEBNext Ultra Directional RNA Library Prep Kit (New England Biolabs, Frankfurt am Main, Germany) was used to prepare strand‐specific libraries. Individually indexed libraries were pooled together and sequenced with single‐end reads (100 bp) on two runs of Illumina NovaSeq S1 at the Vienna Biocenter Core Facilities (VBCF; https://www.viennabiocenter.org/facilities/). All raw read data have been uploaded to NCBI and can be found under BioProject ID PRJNA760819.

### Differential expression analyses

After demultiplexing using BamIndexDecoder v.1.03 (available from http://wtsi-npg.github.io/illumina2bam/#BamIndexDecoder), bam files were converted to fastq using samtools v.1.3 (Li et al., 2009) and quality and adapter trimmed using trimmomatic v.0.36 (Bolger et al., 2014). The individual samples were aligned against the reference genome for *Heliosperma pusillum* v.1.0 (Szukala et al., 2023) using the available gff file for gene annotations and STAR v.2.6.0c (Dobin et al., 2013). A table of counts was produced using FeatureCounts v.2.0.3 from Rsubread package (Liao et al., 2014) including only uniquely mapping reads. After filtering count matrices retaining genes with an average count per million higher than 1, data normalization and differential expression (DE) analyses were performed using the Bioconductor package EdgeR v.3.24.3 (Robinson et al., 2010) implementing a generalized linear model of the type expression = pair + altitude + ecotype + pair*altitude*ecotype to account for the effects of the covariates altitude, ecotype pair and ecotype on gene expression. Gene‐wise dispersion was estimated over all genes using the *estimateDisp()* function and specifying *robust = T* to robustify the estimation against potential outliers. We fitted a gene‐wise negative binomial generalized log‐linear model (EdgeR function *glmFit*), again with the option *robust = T* to decrease the informativeness of outlier genes. A likelihood ratio test (EdgeR function *glmLRT*) was used to test for DE genes and the significance was adjusted using Benjamini–Hochberg correction of *P*‐values to account for multiple testing. Spearman correlation tests between gene expression changes at different altitudes were also performed.

First, we looked for DE genes between ecotypes in one environment and across both environments (i.e., *constitutive expression divergence*). Second, we aimed to detect plastic expression changes due to the component altitude in each of the ecotypes in each pair. We checked, which genes are DE between altitudes in each ecotype, and additionally identified the genes showing a minimum mean fold change (FC) in expression of 1.5 across biological replicates when the growing environment is changed. The FC threshold was set to detect genes showing a strong association with the environmental change. Finally, we tested the statistical significance of the overlap between lists of DE genes using the genes retained after trimming low counts as background and the hypergeometric test of the Bioconductor package SuperExactTest (Wang et al., 2015).

### Biological interpretation of DE genes

To retrieve functional annotations of the genes, we updated the functional annotations of the gene models for the reference genome v.1.0 for *Heliosperma pusillum* (Szukala et al., 2023) by blasting against the latest *Arabidopsis thaliana* database using Blast2GO v.5.2.5 (Götz et al., 2008). Fisher's exact tests implemented in the Bioconductor package topGO v.2.34.0 (https://bioconductor.org/packages/release/bioc/html/topGO.html) were used to identify significantly overrepresented functions (adjusted *P* < 0.05).

### Detecting signatures of ecotype divergence in DE gene

Szukala et al. (2023) investigated signatures of high *F*_ST_ in the same ecotype pairs as in the present study to detect outlier genes potentially under divergent selection. Briefly, per‐locus *F*_ST_ was estimated in ANGSD to account for low coverage values in DE genes and using the folded site frequency spectrum to compute the Bathia *et al*. (2013) *F*_ST_ estimator (refer to publication for more details). Here, we looked for DE genes that carry variant sites falling in the top 5% of the *F*_ST_ distribution to clarify whether a proportion of DE genes also carries signatures of selection at the level of DNA sequence variation. Further, we used the hypergeometric test of the Bioconductor package SuperExactTest (Wang et al., 2015) to test whether the amount of genes carrying signatures of selection falls within or exceeds chance expectations based on the amount of DE genes and genes carrying divergence outliers.

### Detection of population‐wise private alleles

We sorted mapped files according to the mapping position and marked and removed duplicates using Picard v.2.9.2 (https://broadinstitute.github.io/picard/). Variant calling was then performed following standard practices for RNA as implemented in GATK v.4.1.8.1 (Van der Auwera & O'Connor, 2020). First, reads with Ns in the CIGAR string were split using the split'N'trim function and overhangs were trimmed. HaplotypeCaller was used to call variants with the option *‐ERC GVCF*. Subsequently, multiple samples in gvcf format were merged using the GenomicsDBImport utility with the *‐L* option to operate in parallel on multiple genomic intervals. Finally, we used GenotypeGVCFs to perform joint genotyping. We filtered the obtained vcf file first using the vcfallelicprimitives modality implemented in vcflib v.1.0.2 (https://github.com/vcflib/vcflib) with the options *‐‐keep‐info ‐‐keep‐geno* to split multiple nucleotide polymorphisms (MNPs) into multiple SNPs. VCFtools v.0.1.16 (Danecek et al., 2011) was used to keep only high‐quality biallelic SNPs with the options ‐*‐max‐alleles* 2 *‐‐min‐alleles* 2 *‐‐minDP* 4 *‐‐minGQ* 20 *‐‐minQ* 30 *‐‐remove‐indels*. Additionally, we filtered using the *‐‐max‐missing* 1 option to discard all loci with missing genotypes and test for consistency of the results when missingness was not allowed. To detect population‐wise private alleles we used the vcf‐contrast module of VCFtools with the options *‐n ‐f ‐d* 5 and specifying the population samples using *‐‐indv*. Raw numbers of private alleles per population were divided by the number of samples in each ecotype and population (i.e., 10 individuals per ecotype and pair—including plants reared in either environment—except for the montane ecotype of pair 3 with seven individuals). Finally, VCFtools was run on the output files of vcf‐contrast with the option *‐‐freq* and specifying the population samples using *‐‐indv* to obtain major and minor allele frequencies for the private alleles.

## AUTHOR CONTRIBUTIONS

OP, BF, and PS conceived and designed the study. BF and PS performed the sampling of seeds. OP and PS performed the transplantation experiments. AS conducted bioinformatics and statistical analyses, with contribution from CB for visualization of Figure 2. AS first drafted the manuscript and all authors revised and approved the manuscript.

## CONFLICT OF INTEREST

The authors declare no conflicts of interest.

### OPEN RESEARCH BADGES

This article has earned an Open Data badge for making publicly available the digitally‐shareable data necessary to reproduce the reported results. The data is available at https://www.ncbi.nlm.nih.gov/bioproject/PRJNA760819/; https://github.com/aglaszuk/Heliosperma_plasticity_parallelEvol.

## Supporting information


**Figure S1.** Main trends in normalized gene expression counts. Principal component analysis of normalized gene expression counts. Visualization of the components 1–8. Circles represent the ecotype pair 1 and squares the ecotype pair 3. Green‐ and brown‐filled symbols show the montane and alpine ecotypes, respectively, while green and brown symbol margins represent the low and high growing sites, respectively.
**Figure S2.** Population private alleles and their minor allele frequency (MAF). (a) Total amount of private alleles by population after normalization by sample size and considering only biallelic SNPs (no missing data allowed). (b) MAF of private alleles by population. Bars represent the standard deviation.
**Figure S3.** Per population private allele statistics when allowing missing data.

**Table S1.** Constitutive genes always DE between ecotypes regardless of the environment and shared by both ecotype pairs are reported with UniProt database gene and protein IDs, GO IDs, and term annotations, expression logFC, logCPM, and adjusted *P*‐value. The IDs in bold are genes that were found to be DE also in a previous common garden experiment in Szukala et al. (2023).
**Table S2.** GO terms enriched in genes constitutively DE between ecotypes regardless of the environment in pair 1.
**Table S3.** GO terms enriched in genes constitutively DE between ecotypes regardless of the environment in pair 3.
**Table S4.** GO terms enriched in genes DE between altitudes in the montane ecotype of pair 1.
**Table S5.** GO terms enriched in genes DE between altitudes in the montane ecotype of pair 3.
**Table S6.** GO terms enriched in genes DE between altitudes in the alpine ecotype of pair 1.
**Table S7.** GO terms enriched in genes DE between altitudes in the alpine ecotype of pair 3.
**Table S8.** DE genes from each comparison carrying Fst outliers and their functional annotation (yellow cells indicate genes occurring in more than one comparison).

## Data Availability

The raw Illumina sequencing data are deposited on NCBI SRA and can be found under BioProject ID PRJNA760819. The table of raw counts and the lists of differentially expressed genes can be found under this gitHub repository: https://github.com/aglaszuk/Heliosperma_plasticity_parallelEvol
