# Supplementary material for: Parallel adaptation to lower altitudes is associated with enhanced plasticity in Heliosperma pusillum (Caryophyllaceae)
Source: Plant J. 2023 Jun 21;115(6):1619–32. doi: 10.1111/tpj.16342 (PMC10952512; doi:10.1111/tpj.16342)
Supplement: Supplementary file 1 — Figure S1. Main trends in normalized gene expression counts. Principal component analysis of normalized gene expression counts. Visualization of the components 1–8. Circles represent the ecotype pair 1 and squares the ecotype pair 3. Green‐ and brown‐filled symbols show the montane and alpine ecotypes, respectively, while green and brown symbol margins represent the low and high growing sites, respectively. Figure S2. Population private alleles and their minor allele frequency (MAF). (a) Total amount of private alleles by population after normalization by sample size and considering only biallelic SNPs (no missing data allowed). (b) MAF of private alleles by population. Bars represent the standard deviation. Figure S3. Per population private allele statistics when allowing missing data. [file TPJ-115-1619-s001.pdf]

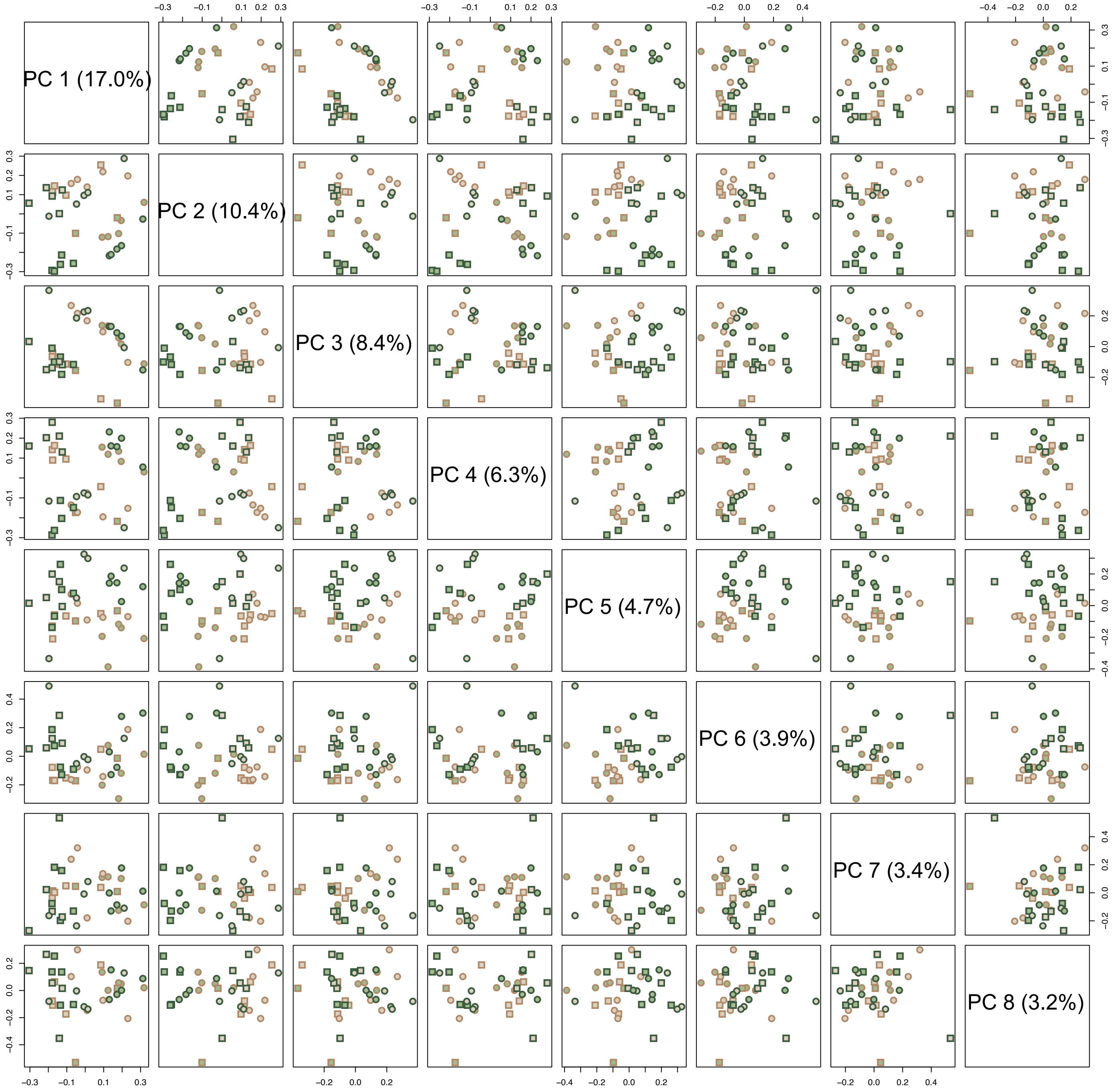

Supplementary Figure S1. Main trends in normalized gene expression counts. Principal component analysis of normalized gene expression counts. Visualization of the components 1 to 8. Circles represent the ecotype pair 1, squares the ecotype pair 3. Green- and brown-filled symbols show the montane and alpine ecotypes, respectively, while green and brown symbol margins represent the low and high growing sites, respectively.

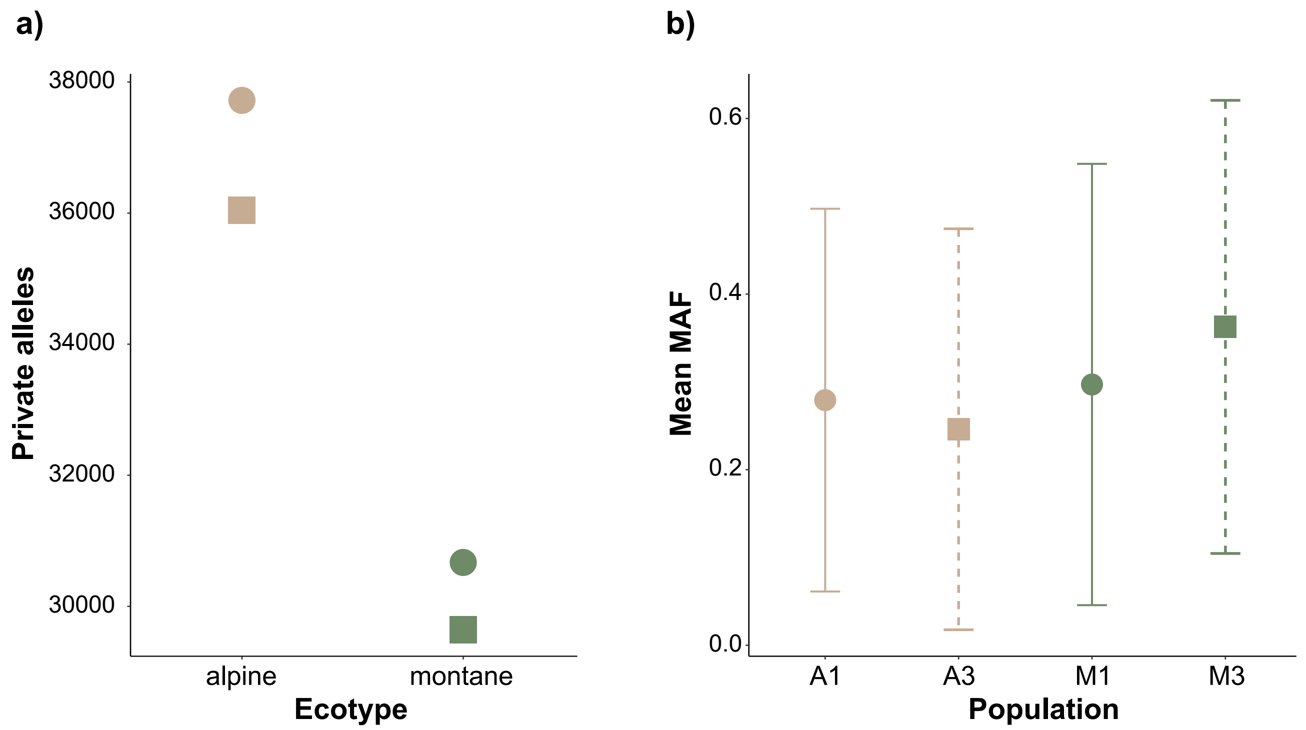

Supplementary Figure S2. Population private alleles and their minor allele frequency (MAF). **(a)** Total amount of private alleles by population after normalization by sample size and considering only biallelic SNPs (no missing data allowed). **(b)** MAF of private alleles by population. Bars represent the standard deviation.

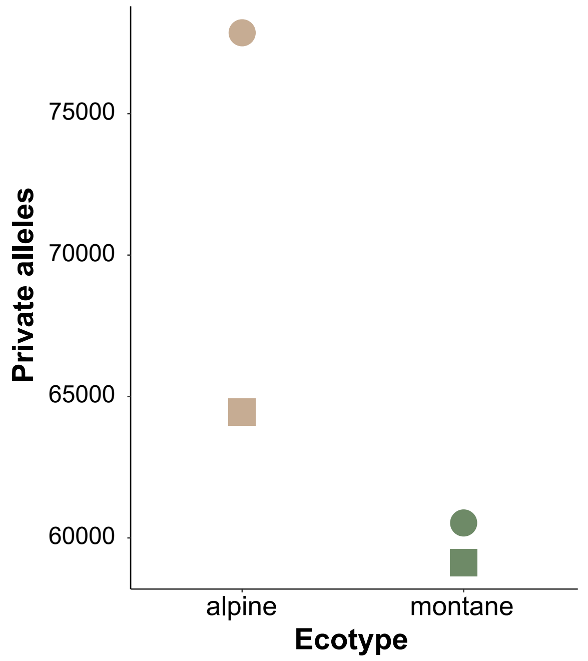

Supplementary Figure S3. Per population private allele statistics when allowing missing data.
